# 脑转移瘤免疫相关疗效评价标准的研究进展

**DOI:** 10.3779/j.issn.1009-3419.2021.101.03

**Published:** 2021-02-20

**Authors:** 舒言 盛, 瑛 胡

**Affiliations:** 101149 北京，首都医科大学附属北京胸科医院，北京市结核病胸部肿瘤研究所，肿瘤内科 Department of Medical Oncology, Beijing Chest Hospital, Capital Medical University/Beijing Tuberculosis and Thoracic Tumor Research Institute, Beijing 101149, China

**Keywords:** 脑转移瘤, 免疫治疗, 疗效评价, Brain metastases, Immunotherapy, Response criteria evaluation

## Abstract

脑转移瘤是成人恶性神经系统肿瘤最常见的病因。针对这一部分人群，治疗手段有限，预后不佳。近年来，以程序性死亡受体1（programmed cell death protein 1, PD-1）及程序性死亡受体配体1（programmed cell death protein ligand 1, PD-L1）抑制剂为主的免疫治疗，给恶性肿瘤的治疗模式带来了革新。免疫检查点抑制剂（immune checkpoint inhibitors, ICIs）彻底改变了晚期非小细胞肺癌（non-small cell lung cancer, NSCLC）的治疗模式。ICI在某些驱动基因阴性的NSCLC脑转移瘤治疗中，取得了令人鼓舞的结果。然而，针对脑转移瘤患者的临床研究，不仅相应的临床数据有限，其疗效的评价也缺乏统一标准。本文旨在阐述不同的疗效评价标准及其在临床研究中的应用，比较之间的异同，并对未来发展趋势进行展望。

## Abstract (en)

Brain metastases are the major cause of adult malignant nervous system tumors. For this part of population, treatment options are limited and the prognosis is poor. In recent years, immunotherapy based on inhibitors of programmed cell death protein 1 (PD-1) and programmed cell death receptor ligand 1 (PD-L1), have brought innovation to the treatment of malignant tumors. Immune checkpoint inhibitors (ICIs) have revolutionized the management of advanced non-small cell lung cancer (NSCLC). Encouraging results have suggested that ICIs could be active in selected advanced NSCLC brain metastases with driver-negative patients. However, for patients with brain metastases, not only the corresponding clinical data are limited, but also the evaluation of its efficacy lacks a unified standard. This article aims to review the relevant efficacy evaluation standards and their application in clinical researches, compare the similarities and differences, and look forward to future trends.

## 1 前言

实体瘤脑转移是成人神经系统最常见的恶性肿瘤^[1]^。在恶性肿瘤患者中，脑转移发生率介于8.5%-9.6%之间^[2-4]^。尽管进展期实体瘤脑转移患者的病死率高，但在系统性全身治疗中，针对脑转移患者的临床数据非常有限。由于神经系统特殊的解剖学特点，大分子药物不能透过血脑屏障以达到要求的血药浓度，导致一部分临床试验将这类患者排除在外^[5]^。另一方面，作为实体瘤中枢神经系统转移灶，在接受系统性或局部治疗后，对其疗效的评价，有的根据实体瘤评价标准，也有根据神经系统肿瘤疗效评价标准而进行的。评价方法的选择存在异质性，缺乏统一标准。

以程序性死亡受体1（programmed cell death protein 1, PD-1）及程序性死亡受体配体1（programmed cell death protein ligand 1, PD-L1）抑制剂为代表的免疫治疗近年来取得了里程碑式的进展，在多个恶性肿瘤的治疗中发挥了举足轻重的作用。不同于直接跨过血脑屏障发挥生物活性^[5, 6]^，研究发现免疫检查点抑制剂（immune checkpoint inhibitors, ICI）可通过调节中枢神经系统的免疫微环境而达到抗肿瘤作用^[7, 8]^。其并不依赖于药物与肿瘤细胞转移灶的直接接触，而是通过加强免疫识别，激活中枢神经系统的炎症细胞等机制来发挥作用^[9]^。在黑色素瘤、非小细胞肺癌（non-small cell lung cancer, NSCLC）以及肾透明细胞癌脑转移的治疗中，其有效性及安全性已被多项临床试验及回顾性分析所证实^[10-14]^。然而，一方面脑转移瘤疗效的评价标准不统一; 另一方面，免疫治疗特有的延迟应答现象及治疗所造成的局部炎症反应导致的“假进展”现象，给疗效的评价带来了挑战。本文旨在回顾相关疗效评价标准及其在临床研究中的应用，以期更合理的应用评价标准服务于临床。

## 2 评价标准的概述及各自局限性

判断治疗前后肿瘤形态的变化是评价治疗疗效的一个手段。脑转移瘤作为实体瘤远处转移灶，有相当一部分临床研究采用了实体肿瘤反应评估标准（Response Evaluation Criteria in Solid Tumors, RECIST）来评估脑转移瘤治疗效果^[15, 16]^。该标准以肿瘤最大径作为肿瘤负荷的观察指标，可测量的靶病灶来自于所有受累及的器官，以所有靶病灶的长度总和，作为肿瘤负荷的参考基线。疾病进展定义为：基线病灶长径总和增加≥20%，或出现新病灶。然而对于脑转移瘤，治疗效果的评价不仅取决于靶病灶大小的改变，还与转移瘤压迫造成的神经功能状态变化及类固醇激素用量的变化密切相关。并且疗效的评价易受其他器官转移灶治疗结果的干扰，一定程度上不能客观反映脑转移灶的治疗效果。

基于这些因素，研究人员建立了MacDonald标准^[17]^，起初用于神经胶质瘤疗效的评价，随后逐渐应用至中枢神经系统其他肿瘤。该标准以肿瘤体积的增大为主要测量指标，同时疗效的评价考虑到类固醇激素的应用情况和患者神经功能状态的变化。肿瘤进展定义为：通过对比增强扫描，靶病灶的体积较前增加≥25%。然而随着新型治疗方法的不断涌现，MacDonald标准在使用中也出现它的局限性。比如靶病灶的对比增强不能简单解释为治疗反应，其变化受类固醇激素的使用、局部炎症反应及手术或放射治疗带来的损伤等多种因素影响。同时，该标准对弥漫浸润病变以及缺乏对比增强病灶的评估有着局限性。

面对高级别脑胶质瘤在放疗及替莫唑胺治疗后出现的假性进展现象以及抗血管生成治疗后出现的假性缓解现象给疗效评价造成的挑战。为更准确客观地进行疗效评价，神经肿瘤反应评价（response assessment in neuro-oncology, RANO）工作组联合多学科于2010年公布了针对高级别脑胶质瘤疗效的评价标准（response assessment in neuro-oncology for high grade gliomas, RANO-HGG）^[18]^。该标准通过增强扫描以及重复扫描等方法以达到准确评估治疗效果的目的，后续又扩展应用至脑转移瘤、软脑膜癌、脑膜瘤等其他中枢神经系统肿瘤中。RANO-HGG标准在MacDonald标准基础上，通过二维测量方法，将可测量病灶定义为：通过电子计算机断层扫描（computed tomography, CT）或磁共振成像（magnetic resonance imaging, MRI）增强检查手段，病变两条垂直直径至少 > 10 mm，并在2个或多个轴面上可见，且要求无间隔扫描层厚≤5 mm; 在存在多个病灶的情况下，进行疗效评价时最多可观察5个靶目标病灶。肿瘤进展定义为：与基线病灶相比，靶病灶两条垂直直径的乘积增加≥25%。为避免放射治疗后局部水肿所引起的影像学假性进展改变给结果造成的混淆，明确放射治疗开始后1周-12周内受辐照区域观察到的新病变均不应视为疾病进展，对判断不明确的病例，间隔至少4周再进行重复扫描，必要时借助病理活检以协助诊断。

然而无论是MacDonald标准还是RANO-HGG标准，最初都是针对神经胶质瘤疗效评价制定的。脑转移瘤兼具原发实体瘤及神经系统肿瘤两方面特点，在评价脑转移瘤疗效时，无论采用上述何种评价标准，都有各自的局限性。为了更高质量地评价脑转移瘤治疗效果，神经肿瘤反应评价小组于2015年公布了神经系统脑转移瘤疗效的评价标准（RANO for brain metastases, RANO-BM）^[19]^。该标准根据脑转移瘤的关键特征，在RECIST标准和RANO-HGG标准的基础上，进行了整合并进一步做了完善。RANO-BM标准使用一维方法测量肿瘤大小，要求可测量病灶直径至少为10 mm，允许最多5个病灶作为靶病灶，并在确定疾病缓解或进展时纳入患者的临床功能状态和类固醇激素使用情况作为评价依据。进展定义为：基线病灶长径增加≥20%，且病灶最小绝对值增加5 mm，或者出现新发病灶，或在MRI T2/FLAIR加权像上有明确进展，伴随患者的临床表现明显恶化。考虑到颅内外病灶对系统性治疗的反应可能不一致，RANO-BM标准要求在评价疗效时，将脑转移瘤与颅外病灶的疗效分别进行评估。

Qian等^[20]^将RANO-BM和RECIST标准再次进行了整合，提出了修订版RECIST（modified RECIST, mRECIST）1.1标准，以适应免疫治疗后脑转移瘤疗效的评价。该标准将可测量病灶长径下限放宽至≥5 mm，这一点不同于以往任何标准，从而使更多的患者有机会被纳入临床研究中，仍允许最多5个病灶作为靶目标病灶。研究发现，虽然靶病灶最长径的缩小可能增加了测量误差，但使用薄层MRI技术（扫描层厚≤2 mm），并按要求进行重复扫描确认，可以有效降低假阳性结果的发生几率。该研究指出，针对免疫治疗脑转移瘤患者疗效的评价，mRECIST标准与RANO-BM标准、RANO-HGG及RECIST标准相比，结果表现出良好的一致性，同时可将更多的患者纳入临床研究，带来更多的临床获益。

## 3 免疫治疗后带来的疗效评价挑战及相应标准

经免疫治疗后，影像学常出现延迟反应或治疗导致的类似假性进展的炎症反应等改变，与实体瘤一样，脑转移瘤患者接受免疫治疗后，颅内病灶有可能出现假性进展的表现^[21, 22]^。Cohen等^[23]^报道了1例黑色素瘤脑转移患者在接受了Pembrolizumab治疗11 d后出现了异常的精神症状，MRI显示病灶周围水肿明显恶化，在T1加权像上病灶体积较前明显增大的病例。考虑到靶病灶体积增长的速度无法用疾病进展进行解释，最终进行局部病理活检提示迅速进展的区域由反应性出血、大量的星型细胞、分散的炎症细胞以及CD68阳性的小胶质细胞所构成，这些全部为典型的免疫治疗后反应。

Wolchok等^[24]^总结了经免疫治疗有效可能出现的4种应答情况：①基线病灶快速消退同时不伴有新病灶的产生; ②保持稳定状态（对于一些患者，表现为缓慢稳定的肿瘤总负荷下降）; ③治疗后早期肿瘤负荷增加，随后肿瘤负荷逐渐减小; ④出现新病灶，但总肿瘤负荷减小。传统治疗手段通常是在治疗结束时即开始疗效评价，而免疫治疗疗效评价不仅需要更多评价指标，还需要更长的观察随访时间。免疫相关反应标准（immune-related response criteria, irRC）指出，如果患者临床状态无明显下降，除非后续影像学检查证实病灶进展，否则早期病灶体积的增加或新病灶的出现不能确定进展^[24]^。神经肿瘤免疫治疗反应评价小组纳入RANO工作组既往标准，为包含脑转移瘤在内的接受免疫治疗的神经系统肿瘤患者制定出了iRANO（Immunotherapy Response Assessment for Neuro-oncology）疗效评价标准^[25]^。该标准建议，对于无临床缓解的患者需要给予6个月的免疫治疗，因为即使早期影像学表现为进展，后期仍有可能有明确的临床获益; 出现影像学进展的患者3个月后需复查影像学，并与最初的影像学结果进行比较，以评价治疗后转归。只有在复查影像学证实疾病进展的情况下，方可回顾性地列为疾病进展，并停止免疫治疗; 如果重复扫描显示肿瘤负荷稳定或减少，则应继续治疗。当判断影像学进展存在困难时，建议行组织活检术以协助诊断。iRANO标准针对免疫治疗后可能出现的假进展问题，通过6个月的时间窗以评估真实疗效，避免了潜在的可能获益人群，过早终止治疗。但在临床试验及临床应用中，其执行存在困难，一定程度限制了iRANO标准的推广。有关不同评价标准之间的比较见表 1。

**1:** RECIST 1.1, mRECIST, RANO-HGG, RANO-BM和iRANO标准之间疗效评价标准的对比 Summary of differences between RECIST 1.1, mRECIST, RANO-HGG, RANO-BM and iRANO assessment criteria

	RECIST 1.1^[16]^	mRECIST^[20]^	RANO-HGG^[18]^	RANO-BM^[19]^	iRANO^[25]^
Measureable lesions	≥10 mm in one diameter	≥5 mm in one diameter	≥10 mm in two diameters	≥10 mm in one diameter	≥10 mm in one diameter (in the case of brain metastases)
Target lesions	2	5	5	5	5
Steroids	Not included	Included	Included	Included	Included
CR	Complete disappearance of all lesions for ≥4 weeks	Complete disappearance of all lesions for ≥4 weeks	Complete disappearance of all lesions for ≥4 weeks; no steroid use; clinically stable/improved	Complete disappearance of all lesions for ≥4 weeks; no steroid use; clinically stable/improved	Complete disappearance of all lesions for ≥4 weeks; no new lesions; no steroids; clinically stable/ improved
PR	≥30% decrease in sum of longest diameters, compared to baseline for ≥4 weeks	≥30% decrease in sum of longest diameters compared to baseline for ≥4 weeks	≥50% decrease in sum of bidimensional products compared to baseline for ≥4 weeks; stable/less steroids; clinically stable/improved	≥30% decrease in sum of longest diameterscompared to baseline for ≥4 weeks; stable/less steroids; clinically stable/improved	≥30% decrease in sum of longest diameters of targetlesions for ≥4 weeks; no new lesions; stable or decreasedsteroid dose; clinically stable/improved
PD^*^	≥20% increase in sum of longest diameterscompared to nadir, with minimum absoluteincrease of 5 mmnew lesions	≥20% increase in sum of longest diameterscompared to nadirnew lesions	≥25% increase in sum of bidimensional productscompared to nadir; significant T2 signalincrease; new lesions; clinical status worse	≥20% increase in sum of longest diameterscompared to nadir, with minimum absoluteincrease of 5 mm in onelesion; significant T2 signalincrease; clinical status worse	≥20% increase in the sum of longest diameters of targetlesions; or unequivocal progression of enhancingnon-target lesions; or new lesions; or substantial clinicaldecline
SD	Does not meet criteria for CR, PR, or PD	Does not meet criteria for CR, PR, or PD	Does not meet criteria for CR, PR, or PD	Does not meet criteria for CR, PR, or PD	Does not meet criteria for CR, PR, or PD
^*^Specifically, iRANO recommends confirmation of disease progression on follow-up imaging 3 months after initial radiographic progression if there is no new or substantially worsened neurological deficits that are not due to comorbid events or concurrent medication, and it is 6 months or less from starting immunotherapy. If follow-up imaging confirms disease progression, the date of actual progression should be back-dated to the date of initial radiographic progression. The appearance of new lesions 6 months or less from the initiation of immunotherapy alone does not define progressive disease^[20, 25]^. RECIST: Response Evaluation Criteria in Solid Tumors; mRECIST: modified RECIST; RANO-HGG: response assessment in neuro-oncology for high grade gliomas; RANO-BM: RANO for brain metastases; iRANO: immunotherapy RANO; CR: complete response; PR: partial response; PD: preogressive disease; SD: stable disease.					

## 4 脑转移瘤免疫治疗疗效评价回顾

随着免疫疗法在脑转移瘤治疗中有效性及安全性得到肯定，越来越多的脑转移瘤患者被纳入免疫治疗相关研究。各项临床试验根据其设计的初衷不同，选择各自相适应的研究终点。诸多因素影响着研究终点的选择。总生存期被认为是判定疗效的金标准和最客观的研究终点，然而，在脑转移瘤患者中，无论是总生存期（overall survival, OS）还是无进展生存期（progression-free survival, PFS），其应用均受到一定限制^[26]^。首先，脑转移瘤患者的预后不仅与颅内情况有关，更受原发灶治疗情况及身体一般状况等因素的影响。其次，接受免疫治疗的脑转瘤移瘤患者，在局部进展后，可能接受补救治疗，若以生存为评价指标会混淆免疫治疗的真实效果。目前在已报道的脑转移瘤免疫治疗的临床试验中，Ⅱ期临床试验占大多数，通常选择颅内客观缓解率（intracerebral objective response rate, IORR）或者颅内疾病控制率（intracerebral disease control rate, IDCR）为首要研究终点。除此之外，随着随访时间的延长，部分试验将PFS或OS作为次要研究终点以进一步评估免疫治疗的临床获益。

Margolin等^[10]^于2012发表了针对72例黑色素瘤脑转移患者接受Ipilimumab治疗的Ⅱ期临床研究结果。通过实体瘤诊断标准，以疾病控制率为首要研究终点，入组的72例患者中，51例无精神系统症状，未接受过类固醇激素治疗; 21例在参加试验前有神经系统症状，正在使用类固醇治疗。通过12周的随访观察，51例中有9例达到疾病控制（18%, 95%CI: 8%-31%），而有神经系统症状的21例患者中只有1例（5%, 95%CI: 0.1%-24%）达到首要研究终点。单独评估脑转移灶时，两组患者中分别有12例（24%, 95%CI: 13%-38%）及2例（10%, 95%CI: 1%-30%）达到中枢神经系统内疾病控制。Goldberg等^[27]^在2016年公布了使用Pembrolizumab单药治疗脑转移瘤的一项非随机、Ⅱ期临床研究（NCT02085070）。初期研究分别纳入18例黑色素瘤和18例NSCLC脑转移患者。要求研究对象至少有1个未经治疗、最长径在5 mm-20 mm之间的脑转移灶，且入组患者无精神系统相关症状，不使用类固醇激素治疗。研究人员以mRECIST标准评价脑转移灶疗效，以RECIST 1.1标准评价颅外病灶疗效。首要研究终点为脑转移灶的ORR。在黑色素瘤及NSCLC患者中，分别有4例（22%, 95%CI: 7%-48%）及6例（33%, 95%CI: 14%-59%）达到首要研究终点。2019年公布了该研究中黑色素瘤患者长期随访结果，共纳入23例，其中脑转移灶的ORR为26%，中位PFS和中位OS分别为2个月及17个月^[28]^。2020年公布了该研究NSCLC部分长期随访结果，入组42例NSCLC脑转移患者，在35例PD-L1≥1%患者中，11例达到首要研究终点（29.7%, 95%CI: 15.9%-47.0%），而在7例PD-L1 < 1%患者中，未观察到有客观缓解的情况^[29]^。Tawbi等^[30]^针对黑色素瘤脑转移患者进行的一项开放、多中心、Ⅱ期临床研究，旨在评估Nivolumab联合Ipilimumab的治疗效果。共纳入94例无神经系统症状的黑色素瘤脑转移患者，以mRECIST标准评价脑转移灶疗效，RECIST 1.1标准评价颅外病灶疗效。以颅内DCR为首要研究终点，次要研究终点分别为颅外DCR、总DCR和相对应的PFS以及OS。94例患者的中位随访时间为14个月，其中颅内DCR为57%（95%CI: 47%-68%），而颅外DCR为56%（95%CI: 46%-67%）。Flippot等^[13]^开展的一项Ⅱ期针对肾透明细胞癌脑转移患者接受免疫治疗的研究，共入组73例患者，以mRECIST标准为疗效评价标准，以未接受过局部治疗患者的颅内客观缓解率为首要研究终点。39例既往未接受过脑转移瘤的治疗，颅内ORR为12%，颅内中位PFS为2.7个月（95%CI: 2.3-4.6）。而34例既往接受过局部治疗的脑转移患者其颅内中位PFS为4.8个月（95%CI: 4.0-8.0）。OAK研究是一项Ⅲ期、多中心、随机对照研究，以既往接受铂类为基础的化疗后再进展的NSCLC患者为研究对象，比较Atezolizumab和多西他赛疗效的临床试验^[31]^。2019年，Gadgeel等^[32]^报道了该研究中既往接受过治疗的脑转移患者的疗效评价结果。两个治疗组中，分别有将近14%的研究对象（Atezolizumab组61/425，多西他赛组62/425）为NSCLC脑转移患者。依据RECIST 1.1标准，以OS为首要研究终点。无论既往治疗情况如何，在有神经系统症状及无症状病例中，Atezolizumab组的中位生存均优于多西他赛组（16.0个月 *vs* 11.9个月，HR=0.74;95%CI：0.49-1.13）（13.2个月 *vs* 9.3个月，HR=0.74;95%CI：0.63-0.88）。

## 5 不同评价标准一致性的比较

对脑转移瘤疗效的评价目前尚无统一标准，不同评价方法之间有相互涵盖之处，也有各自独立的特点。我们关心不同标准的选择是否会让结果产生混淆，导致对药物疗效的评价产生偏倚。

Qian等^[20]^将mRECIST标准和RANO-BM、RECIST 1.1及RANO-HGG标准做了比较，以NCT02085070入组患者为研究对象。以颅内ORR为首要研究终点，分别使用4种评价方法评估脑转移瘤疗效。结果显示，4种标准的结果表现出较高的一致性，与RECIST 1.1和RANO-BM标准相比，将可测量病灶长径放宽至≥5 mm，可多入组13例患者，其中有5例获得了持久应答。与RANO-HGG标准相比，使用mRECIST标准评估脑转移瘤免疫治疗疗效，可多入组19例患者，其中8例获得持久应答，使颅内ORR由12%提高到28%。Le Rhun等^[33]^对62例黑色素瘤脑转移患者进行了回顾性分析，其中10例接受了单药免疫治疗，20例接受了立体定向放疗（stereotatic radiosurgery treatment, SRT）或者SRT联合全身系统治疗，32例接受免疫联合SRT治疗，分别使用RECIST、RANO及iRANO标准评价三组患者的治疗疗效。结果显示三种标准在ORR方面疗效评价结果是一致的，在未进行MRI扫描的患者中，使用RANO标准相比使用RECIST标准，可以更早期确定疾病进展。而对于疾病进展的患者，在使用iRANO标准评价疗效时，因为要求3个月后复查影像学检查结果，结论的产生较其他标准比有滞后现象。

## 6 结语与展望

尽管免疫治疗在脑转移患者中的疗效已取得肯定的结果，其有效性及安全性已被相关临床试验及回顾性研究所证实。但是目前临床研究对疗效评价标准的使用不统一。在脑转移瘤的免疫治疗中，推行使用合理、一致性高的评价标准，有助于标准化疗效监测的推广，促进免疫治疗在脑转移治疗的应用。此外，临床研究终点，根据试验设计目标不同，会有不同的选择。前期已报道的临床研究，多为明确免疫治疗在脑转移瘤患者中的有效性，首要研究终点的选择，以ORR或DCR为主。但随着免疫治疗地位的提高及患者生存预后的不断改善，根据脑转移瘤特点，以神经系统症状、认知功能及生活质量为研究终点的试验将成为可能，相关评价标准有待进一步完善。最后，新的成像技术为免疫治疗疗效的评价创造了新的契机，其在脑转移瘤方面的应用前景值得期待^[34]^。
